# Population pharmacokinetics and model-informed precision dosing of busulfan in Chinese pediatric allogeneic hematopoietic stem cell transplantation: a prospective real-world cohort study

**DOI:** 10.3389/fphar.2026.1974352

**Published:** 2026-10-26

**Authors:** Yewei Chen, Wenjin Jiang, Ping Wang, Ling Yu, Xiaowen Qian, Guangfei Wang, Jie Cai, Mengdi Hu, Xiaowen Zhai, Zhiping Li

**Affiliations:** 1 Department of Pharmacy, Children’s Hospital of Fudan University, National Children’s Medical Center, Shanghai, China; 2 Department of Hematology, Children’s Hospital of Fudan University, National Children’s Medical Center, Shanghai, China

**Keywords:** busulfan, hematopoietic stem cell transplantation, pediatric patients, population pharmacokinetics, precision dosing

## Abstract

**Background:**

Busulfan is a cornerstone of conditioning regimens for pediatric allogeneic hematopoietic stem cell transplantation (HSCT), yet its narrow therapeutic index and high pharmacokinetic variability necessitate individualized dosing. This study aimed to develop and evaluate a population pharmacokinetic (PopPK) model for busulfan in Chinese pediatric allogeneic HSCT recipients, characterize the exposure-response relationship, and establish a model-informed precision dosing (MIPD) strategy for clinical implementation.

**Methods:**

This prospective, single-center study was conducted at Children’s Hospital of Fudan University between August 2020 and January 2026. Plasma busulfan concentrations were quantified using a validated HPLC-MS/MS. PopPK analysis was performed using nonlinear mixed-effects modeling. The association between busulfan exposure (AUC_0–6h_) and clinical outcomes was evaluated using logistic regression. Monte Carlo simulations were performed to derive an age- and body weight-stratified dosing regimen, and a closed-loop MIPD workflow was developed for clinical implementation.

**Results:**

A total of 290 pediatric HSCT recipients contributed 1543 busulfan concentration measurements. A two-compartment model with first-order elimination adequately characterized busulfan pharmacokinetics. Body weight and age were significant determinants of clearance. For a 70 kg reference individual, the population estimates of clearance and central volume of distribution were 12.4 L/h and 40.8 L, respectively. The model demonstrated good predictive performance in internal evaluation. Under empirical dosing, only 47.6% of patients achieved the target AUC_0–6h_ of 900–1350 μM min/L. Busulfan exposure (AUC_0–6h_) was not significantly associated with mortality (OR, 1.002; 95% CI, 1.000–1.004; P = 0.069) or acute graft-versus-host disease (OR, 1.001; 95% CI, 1.000–1.002; P = 0.161). Monte Carlo simulations supported an age- and body weight-stratified initial dosing regimen. A closed-loop MIPD workflow was established in which the initial dose is selected according to the simulated dosing regimen, followed by therapeutic drug monitoring and pharmacist-physician collaborative dose adjustment when AUC_0–6h_ falls outside the prespecified target range.

**Conclusion:**

A PopPK model with body weight and age as key determinants of busulfan clearance was developed and evaluated in a large real-world cohort of Chinese pediatric allogeneic HSCT recipients. The proposed MIPD framework provides a practical approach for optimizing busulfan therapy in this population.

## Introduction

Busulfan is a key component of conditioning regimens for allogeneic hematopoietic stem cell transplantation (HSCT) but has a narrow therapeutic index (Lawson et al., 2021; Chen et al., 2018; Paci et al., 2014). For intravenous busulfan in pediatric patients, regulatory recommendations include a target AUC_0–6h_ of 900–1350 μM min according to the US Food and Drug Administration (FDA) and 900–1500 μM min according to the European Medicines Agency (EMA) (Palmer et al., 2016). AUC_0–6h_ was defined as busulfan exposure during the first 6 h following administration of the first dose. Excessive busulfan exposure has been associated with severe toxicities, including hepatic veno-occlusive disease (VOD), pulmonary toxicity, seizures, and graft-versus-host disease (GVHD), whereas inadequate exposure may compromise engraftment and disease control (Salman et al., 2020; Bartelink et al., 2016; Dirksen et al., 2019). However, a substantial proportion of pediatric patients fail to achieve the target exposure following conventional initial dosing (Jansing et al., 2021; Neroutsos et al., 2021). This challenge is particularly relevant in children because of pronounced interindividual variability in drug disposition across developmental stages, underscoring the need for individualized dosing strategies.

Population pharmacokinetic (PopPK) modeling provides a quantitative framework for characterizing pharmacokinetic variability and integrating patient-specific characteristics, dosing history, and therapeutic drug monitoring data to support individualized therapy (Berezowska et al., 2025; Sheiner et al., 1972). Although several PopPK models have been developed for pediatric HSCT recipients in Caucasian, Japanese, and Korean populations (Savic et al., 2013; Ishiwata et al., 2018; Johnson et al., 2008; Long-Boyle et al., 2015; Rhee et al., 2017; Solans et al., 2020; Hoogd et al., 2020; Veal et al., 2012; Paci et al., 2012; Balis et al., 2025; Neroutsos et al., 2022), the past few years have witnessed a growing body of research specific to Chinese pediatric cohorts. These studies, however, have reported heterogeneous estimates of busulfan clearance. Yuan et al. reported a clearance of 11.08 L/h normalized to a 70-kg adult in 76 Chinese pediatric patients (Yuan et al., 2021), whereas Du et al. reported a lower estimate of 7.71 L/h in a cohort of 128 patients (Du et al., 2022). More recently, our group developed a semi-mechanistic PopPK model incorporating glutathione S-transferase (GST) activity in 65 Chinese pediatric allogeneic HSCT recipients, identifying normal fat mass, age-dependent maturation, and GST enzyme activity as important contributors to busulfan pharmacokinetic variability (Cao et al., 2025). Nevertheless, that previous work was limited by its moderate sample size, and more importantly, did not evaluate the exposure-outcome relationship or propose model-informed dosing regimens based on routinely available clinical data.

To address these gaps, we developed and evaluated a PopPK model of busulfan using a large real-world cohort of Chinese pediatric allogeneic HSCT recipients. Thw objectives of this study were to (1) characterize busulfan pharmacolinetics and identify cilinically relevant covariates; (2) evaluate the association between busulfan exposure, expressed as AUC_0–6h_, and clinical outcomes; and (3) derive an optimized initial dosing regimen using Monte Carlo simulations and establish a closed-loop model-informed precision dosing (MIPD) framework for clinical implementation.

## Methods

### Patients and data collection

A prospective, single-center pharmacokinetic (PK) study was conducted at Children’s Hospital of Fudan University between August 2020 and January 2026. Patients younger than 18 years who received intravenous busulfan as part of an allogeneic HSCT conditioning regimen were eligible for inclusion. Patients were excluded if busulfan PK data were unavailable because of unsuccessful or inadequate sample collection. The study was approved by the Ethics Committee of Children’s Hospital of Fudan University (ethics approval number: 2020-271) and was conducted in accordance with the principles of the Declaration of Helsinki. Written informed consent was obtained from parents or legal guardians and, when appropriate, from the patients themselves. Clinical trial number: not applicable.

Demographic, laboratory, and clinical data were collected for each patient, including gender, body weight (BW), age, height (HT), body surface area (BSA), alanine aminotransferase (ALT), aspartate aminotransferase (AST), blood urea nitrogen (BUN), direct bilirubin (DBIL), total bilirubin (TBIL), serum creatinine (Scr), creatinine clearance rate (CLCR), albumin (ALB), and concomitant medications. Creatinine clearance was estimated using the Schwartz formula, with *k* values of 0.45 for infants younger than 1 year, 0.55 for children aged 1–13 years, 0.7 for males aged 13–18 years, and 0.55 for females aged 13–18 years (Schwartz et al., 1987).

### Busulfan dosing and pharmacokinetic sampling

All patients received intravenous busulfan (Busulfex; Otsuka) at doses ranging from 0.5 to 1.5 mg/kg administered as 2-h infusion every 6 h for a total of 12 doses. Busulfan was administered in combination with cyclophosphamide, fludarabine, cladribine, cytarabine, or etoposide. Levetiracetam or phenobarbital was initiated 1 day before the first busulfan dose for seizure prophylaxis. GVHD prophylaxis was achieved using cyclosporine or tacrolimus in combination with mycophenolate mofetil and methotrexate. Heparin and alprostadil were administered for VOD prophylaxis. Caspofungin or micafungin was used for antifungal prophylaxis.

The pharmacokinetic sampling strategy evolved during the study period. Before November 2021, blood samples were collected at 2, 2.5, 3, 4, and 6 h after the start of the first busulfan infusion, with additional trough samples collected before the sixth or 12th dose. Following completion of the 12th dose, terminal elimination samples were collected according to one of two schedules: 0, 2, 4 and 8 h or 0, 2, 6 and 12 h. After November 2021, a sparse sampling strategy was implemented, with samples collected at 2, 3, 4 and 6 h after the start of the first infusion.

### Determination of busulfan concentration

Plasma busulfan concentrations were quantified using a validated high-performance liquid chromatography-mass spectrometry (HPLC-MS) method. The calibration curve was linear over a concentration range of 10–5000 ng/mL, with a lower limit of quantification of 10 ng/mL.

### Population pharmacokinetic analysis

#### Model development

Population PK analysis was performed using nonlinear mixed-effects modeling with NONMEM software (version 7.5.1, Icon Development Solutions). Pirana (version 2.9.4, http://www.pirana-software.com) was used as an interface for PsN (version 4.9.0, https://uupharmacometrics.github.io/PsN/) (Keizer et al., 2013). Graphical analyses were performed using R software (version 4.5.0, http://www.r-project.org/). Parameter estimation was conducted using the first-order conditional estimation method with interaction (FOCE-I) (Bauer, 2019). Both one- and two-compartment structural models were evaluated as candidate base models. Interindividual variability (IIV) was characterized using an exponential model (Equation 1).

$$P_{i}=\overset{⌢}{P}\times \exp\left(\eta_{i}\right)$$ (1)

Where *P*_*i*_ is the parameter estimate for the *i*th individual, $\overset{⌢}{P}$ represents the typical population value, and $\eta_{i}$ is an inter-individual random effect assumed to follow a normal distribution with mean zero and variance $\omega^{2}$ . Several residual unexplained error variability models were evaluated, including proportional, additive, and combined proportional-additive error models (Equation 2).

$$C\_obs,ij=C\_pred,ij\times \exp\left(\varepsilon_{1}\_ij\right)+\varepsilon_{2}\_ij$$ (2)

Where C_obs,ij is the *j*th observed concentration for the *i*th individual, C_pred,ij is the corresponding individual predicted concentration, and $\varepsilon_{1}\_ij$ and $\varepsilon_{2}\_ij$ are independent random variables each following a normal distributions N (0, σ_1_^2^) and N (0, σ_2_^2^), respectively.

Body size (body weight) and maturation (age) are known to significantly influence busulfan pharmacokinetics in pediatric patients (Savic et al., 2013; Long-Boyle et al., 2015; Solans et al., 2020), and were thus incorporated *a priori* into the model. The effect of body weight on clearance was initially described by allometric scaling using Equation 3.

$$CL=CL_{std}\cdot {\left(\frac{BW}{70}\right)}^{k1}$$ (3)

Where *CL* is the clearance, *CL*_*std*_ represents the typical clearance for a 70-kg reference adult, *BW* is individual body weight, and *k*_*1*_ is the exponent. Based on the well-established allometric relationship between body size and drug clearance, k_1_ was fixed to 0.75. Maturation effects on clearance were evaluated using a sigmoidal *E*max function (Equation 4).

$$CL=CL_{p}\cdot \left(\frac{1}{1+{\left(\frac{Age}{TM50}\right)}^{-\gamma }}\right)$$ (4)

Where *CL*_*p*_ is the population parameter estimate for clearance, *TM*_*50*_ is the age at which clearance reaches 50% of the mature value, and *γ* is the Hill coefficient determining the steepness of the maturation curve. Continuous covariates were assessed using an exponential model (Equation 5), whereas dichotomous covariates were incorporated using a categorical variable equation (Equation 6).

$$P_{i}=\overset{⌢}{P}\times {\left(Cov_{i}/Cov_{median}\right)}^{\theta_{\text{cov}}}$$ (5)

$$P_{i}=\overset{⌢}{P}\times \left(1+\theta_{cat}\times Cov_{i}\right)$$ (6)

Where *P*_*i*_ is the parameter estimate for the *i*th individual, $\overset{⌢}{P}$ is the population parameter estimate, $Cov_{i}$ is the covariate of the *i*th individual, $Cov_{median}$ is the population median of the covariate, and $\theta_{\text{cov}}$ or $\theta_{cat}$ represents the estimated coefficient.

For nested models, covariate selection followed a forward inclusion and backward elimination procedure based on changes in the objective function value (OFV). A reduction in OFV of 3.84 (p < 0.05) served as the criterion for forward inclusion, while a stricter criteria (an increase in OFV of 10.83, p < 0.001) was applied for backward elimination. When two or more covariates significantly improved the model fit, the covariate that led to the greatest reduction in OFV was retained. For non-nested models, the Akaike information criterion (AIC) and Bayesian information criterion (BIC) were utilized to identify superior models, with lower AIC and BIC values indicating better model fit. After the structural and covariate models were established, the covariance between CL and V_1_ was tested using a block omega matrix. The covariance term was retained in the final model based on improved model fit.

#### Model evaluation

The model was evaluated using goodness-of-fit diagnostics, including plots of observed concentrations versus population predictions (DV vs. PRED), DV versus individual predictions (DV vs. IPRED), conditional weighted residuals (CWRES) versus time, and CWRES versus PRED.

Model stability was assessed by non-parametric bootstrap (Ette et al., 2003). One thousand datasets were generated by re-sampling from the original dataset. The model was considered stable if the parameter estimates and their corresponding 95% confidence intervals from the bootstrap closely agreed with those obtained from the original model.

A prediction-corrected visual predictive check (pcVPC) was performed to evaluate the predictive performance of the model based on 1000 simulations (Bergstrand et al., 2011). The observed concentration-time profiles were overlaid with the median values along with the 5th and 95th percentiles of the simulated data. The model was deemed to have satisfactory predictive performance if the majority of observed data points fell within the simulated fifth-95th percentile intervals.

For internal evaluation, an internal evaluation cohort comprising 80 HSCT patients were used. Population-predicted concentrations were obtained using *post hoc* estimation in NONMEM (MAXEVAL = 0) (Johnston et al., 2024) and compared with observed concentrations. Predictive performance was assessed using median prediction error (MDPE) and median absolute prediction error (MDAPE), calculated as follows (Equation 7; Equation 8)

$$MDPE=median\left(\frac{PRED_{i}-OBS_{i}}{OBS_{i}}\times 100\%,i=1,\cdot \cdot \cdot ,n\right)$$ (7)

$$MDAPE=median\left(\left|\frac{PRED_{i}-OBS_{i}}{OBS_{i}}\right|\times 100\%,i=1,\cdot \cdot \cdot ,n\right)$$ (8)

where *OBS*_*i*_ is the observed concentration of the *i*th subject, and *PRED*_*i*_ is the population-predicted concentration. The proportions of prediction errors within ±20% and ±30% were also calculated. MDPE values closer to zero, lower MDAPE values and higher proportions within ±20% and ±30% were considered indicative of better predictive performance.

To further assess the robustness and individual-level predictive performance of the final model, leave-one-out cross-validation (LOOCV) was performed. In each iteration, one patient was excluded from the full dataset, the model was refitted using the remaining patients, and the concentrations of the excluded patient were predicted using the refitted model. This procedure was repeated until every patient had been excluded once. MDPE, MDAPE, and the proportion of prediction errors within ±20% were subsequently calculated. LOOCV was performed using the crossval tool in PsN.

#### Assessment of busulfan exposure and clinical outcomes

Individual busulfan pharmacokinetic parameters were obtained by *post hoc* estimation using the POSTHOC option (MAXEVAL = 0) in NONMEM, based on the final population pharmacokinetic model developed in this study. For each patient, the individual predicted concentration-time profile from 0 to 6 h after the first busulfan dose was generated at 0.5-h intervals, and the corresponding AUC_0–6h_ was calculated using the trapezoidal method in GraphPad Prism (Ver. 4.0, GraphPad Software Corporation, San Diego, United States of America). A single AUC_0–6h_ estimate was obtained for each patient. The proportion of patients achieving the target AUC_0–6h_ of 900–1350 μM min was assessed. Associations between AUC_0–6h_ and clinical outcomes, including mortality and acute GVHD, were evaluated using nultivariable logistic regression with adjustment for relevant demographic and transplant-related covariates. All statistical analyses were performed with StataSE 16 (StataCorp LLC, College Station, TX). All tests were two-sided, and *P* < 0.05 was considered statistically significant.

#### Dosing optimization

Monte Carlo simulations were performed using the original dataset of 290 pediatric patients. Patients were stratified into subgroups according to the covariates retained in the final model. Dosages were evaluated across an empirical range of 0.8–1.2 mg/kg. For each dosing scenario, 1000 replications were performed. A regimen was considered clinically effective if ≥ 80% of simulated patients achieved a predicted AUC_0–6h_ within the target range of 900–1350 μM min.

Based on the final PopPK model and the optimized dosing regimen derived from Monte Carlo simulations, a practical closed-loop MIPD workflow was developed to facilitate clinical implementation.

## Results

### Study population

A total of 290 pediatric HSCT recipients contributed 1543 busulfan concentration measurements. The cohort was divided into a model development cohort of 210 patients and an internal evaluation cohort of 80 patients. Both cohorts were derived from the same single-center study population. Patient demographic and clinical characteristics are summarized in Table 1.

**TABLE 1:** Characteristics of pediatric hematopoietic stem cell transplantation recipients.

Characteristics	Model-building dataset		Internal evaluation dataset	
	Mean (±SD)	Median (range)	Mean (±SD)	Median (range)
Malignant disorders	18 (6.0%)			
Non-malignant disorders	272 (93.6%)			
Patients	210 (72.4%)	​	80 (27.6%)	​
Samplings	1223 (79.3%)	​	320 (20.7%)	​
Busulfan dosage (mg/kg)	1.0 (0.2)	1.0 (0.5–1.5)	1.0 (0.2)	1.1 (0.8–1.3)
Busulfan AUC (μM·min)	925 (196)	917 (566-1687)	951 (198)	909 (570-1574)
Gender (boys/girls)	149/61	​	63/17	​
BW (kg)	16.8 (11.6)	12.5 (2.9–70.0)	19.0 (12.7)	13.0 (4.3–55.3)
Age (years)	4.3 (4.0)	2.7 (0.2–17.2)	5.2 (4.8)	3.2 (0.3–17.3)
HT (cm)	96.4 (29.1)	90.0 (52.0–176.0)	102.2 (32.8)	94.5 (56.5–171.0)
BSA (m^2^)	0.7 (0.3)	0.6 (0.2–1.8)	0.7 (0.4)	0.6 (0.3–1.6)
ALT (U/L)	36.3 (68.8)	17.8 (5.5–679.8)	36.9 (60.4)	20.5 (5.9–390.9)
AST (U/L)	52.4 (101.9)	31.3 (9.9–1043.7)	41.5 (37.0)	31.9 (14.3–310.6)
TBIL (μmol/L)	7.1 (13.8)	4.5 (1.3–192.4)	6.4 (4.7)	4.9 (1.5–29.0)
DBIL (μmol/L)	3.2 (11.0)	1.9 (0.4–158.7)	2.5 (2.0)	2.0 (0.7–13.4)
BUN (mmol/L)	3.5 (1.4)	3.3 (0.4–9.8)	3.6 (1.9)	3.4 (0.5–16.9)
SCR (µmol/L)	27.5 (10.4)	24.4 (12.0–75.7)	38.2 (109.6)	24.8 (10.3–1003.4)
CLCR (mL/min/1.73 m^2^)	170.2 (42.3)	164.9 (69.4–348.2)	190.5 (64.5)	181.9 (7.4–478.6)
ALB (g/L)	36.5 (3.8)	36.5 (21.8–46.2)	36.5 (4.1)	36.2 (26.3–47.7)
Events				
Death	21 (7.2%)	​	​	​
Graft-versus-host disease	123 (42.4%)	​	​	​
Graft failure	14 (4.8%)	​	​	​

^*^ BW, body weight; HT, height; BSA, body surface area; ALT, alanine transaminase; AST, aspartate transaminase; TBIL, total bilirubin; DBIL, direct bilirubin; BUN, urea nitrogen; SCR, serum creatinine; CLCR, creatinine clearance rate; ALB, serum albumin.

### Population pharmacokinetic modeling

A two-compartment model significantly improved model fit compared with a one-compartment model (OFV, 14293 vs. 14491; ΔOFV = −198) and was therefore selected as the structural base model (Table 2, Model II). Residual unexplained variability was best described by a proportional error model.

**TABLE 2:** Effect of covariate analysis—impact of each covariate when added sequentially to the model.

Models	Model description	OFV	AIC	ΔAIC	BIC	ΔBIC	ωCL	η-shrinkage (%)
Model I	1-Compartment	14491	14509	0	14555	0	0.69	0.9
Model II	2-Compartment	14293	14335	−174	14442	−113	0.70	0.8
Model III	$CLstd\cdot {\left(BW/70\right)}^{0.75}$ $V\cdot \left(BW/70\right)$	13668	13710	−799	13817	−738	0.35	2.9
Model IV	$CLstd\cdot {\left(BW/70\right)}^{\mathrm{EXP}}$ $V\cdot \left(BW/70\right)$	13709	13769	−740	13923	−632	0.30	4.1
Model V	$CLstd\cdot {\left(BW/70\right)}^{0.75}$ $FMATV\cdot \left(BW/70\right)$	13595	13677	−832	13886	−669	0.29	4.1
Model VI	$CLstd\cdot {\left(BW/70\right)}^{\mathrm{EXP}}$ $FMATV\cdot \left(BW/70\right)$	13592	13701	−808	13977	−578	0.29	4.2

*OFV, objective function value; AIC, akaike information criteria; ΔAIC, changes in akaike information criteria; BIC, bayesian information criterion; ΔBIC, changes in bayesian information criterion; ωCL, inter-subject variability of clearance; CL*std*, the clearance in an adult with a body weight of 70 kg; BW, body weight.

* $FMAT=\frac{1}{1+{\left(\frac{Age}{TM50}\right)}^{-\gamma }}$ , where γ is the Hill coefficient for clearance, and TM50 is the age at which clearance reaches 50% of the mature value.

To account for body size and maturation, several alternative models incorporating body weight and age were evaluated (Table 2, Model III ∼ VI). The model incorporating allometric scaling of body weight on clearance with a fixed exponent of 0.75, together with a sigmoidal maturation function for age, and linear scaling of central volume of distribution (V_1_) with body weight provided the greatest improvement in model performance (Model V). Compared with the base model, this model resulted in substantial reductions in AIC (ΔAIC = −832) and BIC (ΔBIC = −669). Incorporation of these covariates reduced the between-subject variability (BSV) in clearance (ωCL) from 0.70 to 0.29 and that in V_1_ (ω_V1_) from 0.80 to 0.24. All retained covariates passed the backward elimination criteria and no additional covariates significantly improved model performance. Final parameter estimates are presented in Table 3. The inclusion of a covariance term between CL and V_1_ further improved model fit (ΔOFV = −69, df = 1, P < 0.001). The estimated covariance was 0.055, corresponding to a correlation coefficient of 0.661 between the individual random effects for CL and V_1_. The covariance term was therefore retained in the final model.

**TABLE 3:** Parameter estimates of the final busulfan model and bootstrap validation.

Parameter	Final model		Bootstrap n = 1000	
​	Population estimate	RSE (%)	Median	95% CI
Central clearance (L/h): $CL=\theta_{1}*\left(BW/70\right)**0.75*\left(1/\left(1+\left(Age/\theta_{5}\right)**\left(-\theta_{6}\right)\right)\right)$				
θ_1_	12.4	10.4	12.3	10.8–19.9
θ_5_	0.217	36.6	0.237	0.107–2.18
θ_6_	0.6	34.0	0.631	0.241–1.09
Central volume of distribution (L): $V_{1}=\theta_{2}*\left(BW/70\right)$				
θ_2_	40.8	4.2	40.6	36.5–44.5
Inter-compartmental clearance (L/h): $Q=\theta_{3}$				
θ_3_	0.897	27.4	0.927	0.552–1.68
Peripheral volume of distribution and (L): $V_{2}=\theta_{4}$				
θ_4_	1.27	20.8	1.32	0.849–1.99
Inter-individual variability				
CL (%CV)	31.1	16.0	30.8	26.2–36.1
V_1_ (%CV)	26.9	24.0	27.1	21.2–34.4
Cov (CL, V_1_)	0.055	​	​	​
Correlation (CL, V_1_)	0.661	​	0.665	0.49–0.788
Residual error model				
Proportional (%CV)	17.9	12.8	17.6	15.5–20.0

* RSE, relative standard error; CI, confidence interval.

Diagnostic plots demonstrated an excellent model fit (Figure 1). Bootstrap yieded 981 successful convergences among 1000 replicates, supporting the stability of the final model. Median bootstrap parameter estimates were consistent with those obtained from the final model (Table 3). The pcVPC for the final model is presented in Figure 2. The pcVPC demonstrated good agreement between the observed concentration-time profiles and the corresponding simulated distributions, with the observed median and 5th and 95th percentiles generally contained within the corresponding prediction intervals (Figure 2).

**FIGURE 1:**
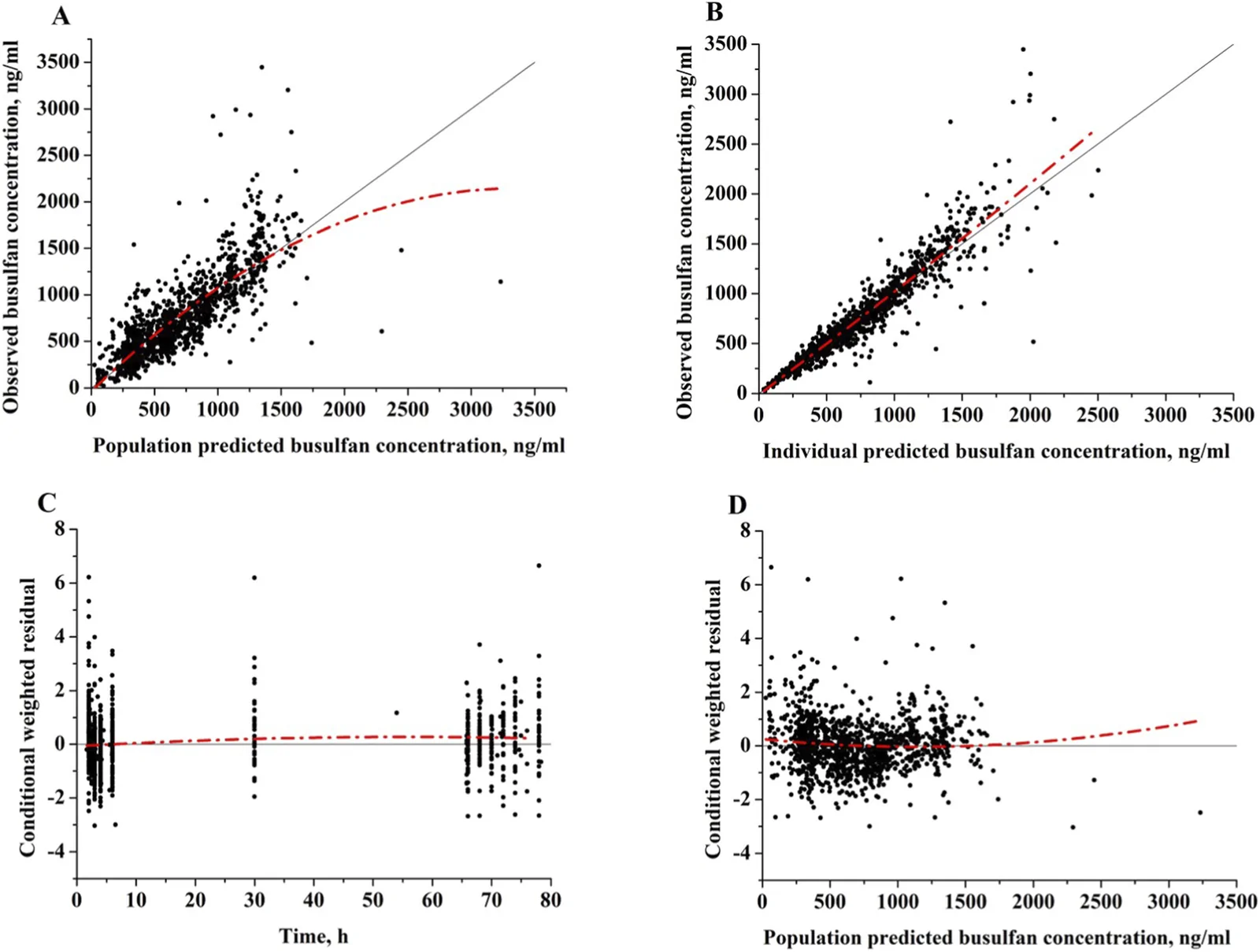
Diagnostic plots of final busulfan model. **(A)** The observed versus population-predicted concentration. **(B)** The observed versus individual-predicted concentration. **(C)** Conditional weighted residual versus time. **(D)** Conditional weighted residual versus the predicted concentration.

**FIGURE 2:**
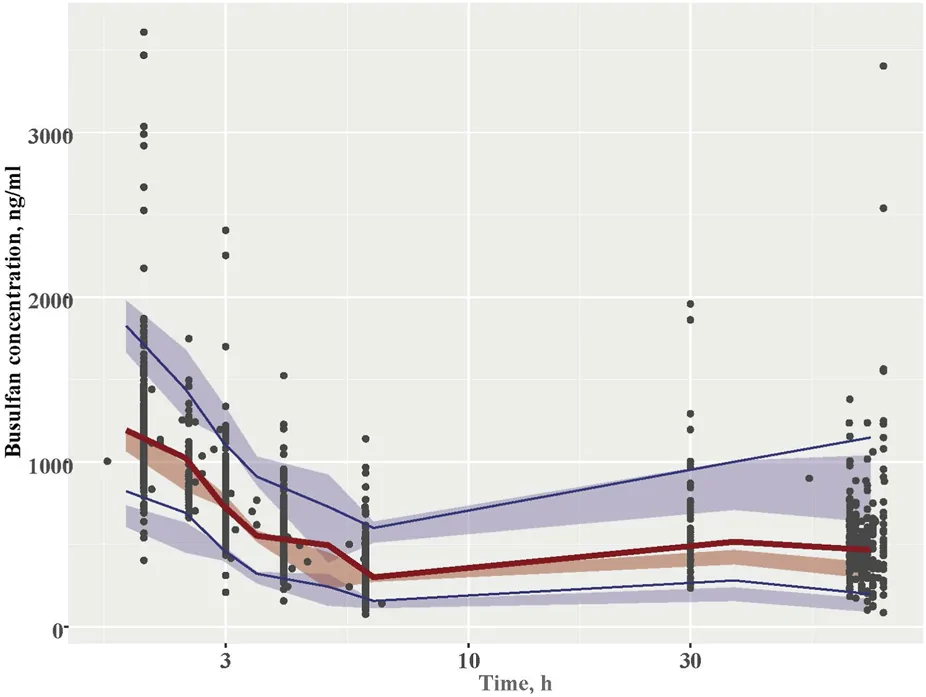
Prediction-corrected visual predictive check of the final pharmacokinetic model. Circles represent the observed concentrations. Solid lines represent the observed median, 5th and 95th percentiles. The shaded areas correspond to the simulation-based 95% confidence intervals.

Internal evaluation further supported the predictive performance of the final model in an internal evaluation cohort of 80 pediatric HSCT recipients. The plot of observed versus population-predicted busulfan concentrations in the evaluation dataset is shown in Figure 3. The MDPE and MDAPE were 0.18% and 17.3%, respectively. The proportions of population prediction error within ±20% and ±30% were 55.6% and 73.8%, respectively, indicating acceptable predictive performance of the final model in the internal evaluation cohort.

**FIGURE 3:**
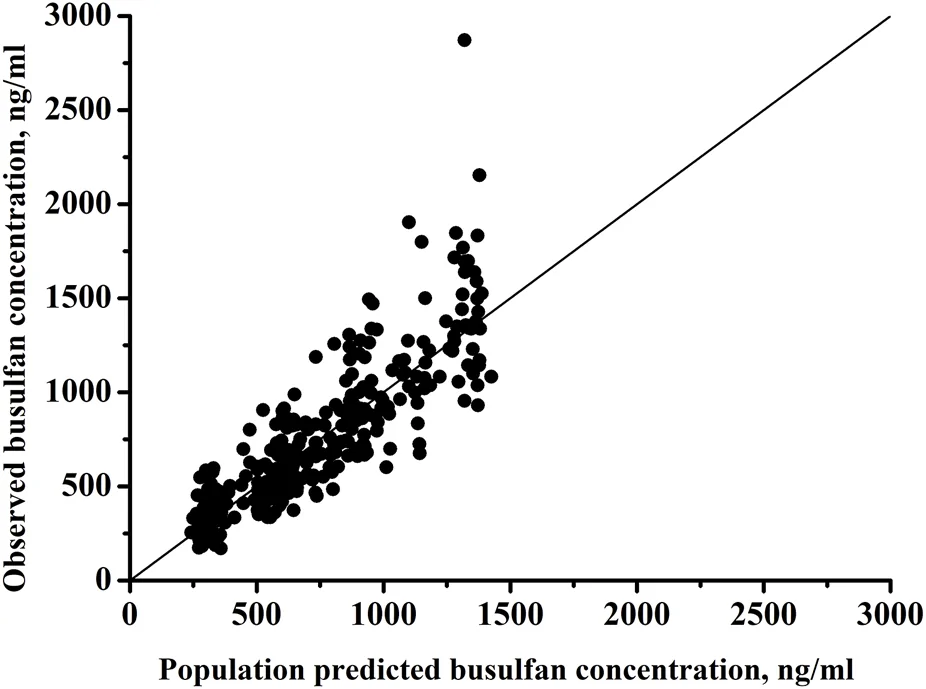
Observed busulfan concentrations versus population-predicted concentrations for the internal evaluation dataset.

LOOCV was subsequently performed to further assess the robustness and individual-level predictive performance of the final model. A total of 290 patients and 1543 observed concentrations were included in the analysis. The model demonstrated an MDPE of −1.72% and an MDAPE of 6.95%, with 88.3% of predictions falling within ±20% of the corresponding observed concentrations. The LOOCV results supported the robustness of the final model and its satisfactory individual-level predictive performance.

### Busulfan exposure and clinical outcomes

Busulfan AUC_0–6h_ demonstrated substantial inter-individual variability, with a median of 904 μM min and a range of 501–1777 μM min. Under empirical dosing, 138 of 290 patients (47.6%) achieved the target range of 900–1350 μM min, whereas 11 patients (3.8%) had supratherapeutic exposure and 141 patients (48.6%) had subtherapeutic exposure.

During follow-up, 21 patients (7.2%) died after HSCT, including 2 deaths attributable to hemorrhagic shock, two to multiple organ failure, and 17 to severe infection. Graft failure occurred in 14 patients (4.8%), VOD in 16 patients (5.5%), and grade I-IV acute GVHD in 123 patients (42.4%). AUC_0–6h_ was significantly higher among non-survivors than survivors. A higher AUC_0–6h_ was also observed among patients who developed acute GVHD compared with those who did not (Figure 4).

**FIGURE 4:**
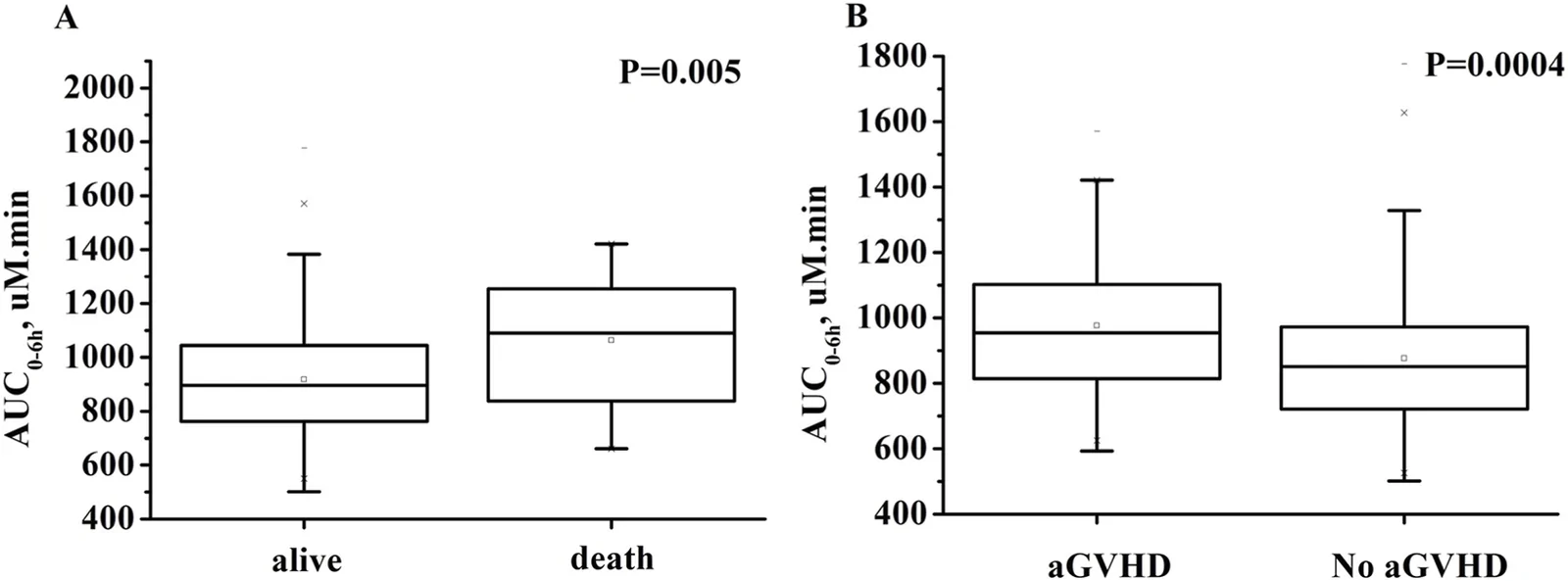
The correlation between busulfan AUC_0–6h_ and clinical outcomes in HSCT patients. Busulfan AUC_0–6h_ against **(A)** occurrence of death; **(B)** occurrence of acute GVHD.

In multivariable logistic regression analysis (Table 4), donor source was independently associated with mortality. Compared with peripheral blood, cord blood transplantation was associated with a higher odds of mortality (OR, 3.574; 95% CI, 1.181–10.817; *P* = 0.024). Busulfan AUC_0–6h_ was not signicantly associated with mortality (OR, 1.002; 95% CI, 1.000–1.004; *P* = 0.069). Gender, age, body weight, disease type, and HLA disparity were not significantly associated with mortality. For acute GVHD, busulfan AUC_0–6h_ was not significantly associated with the outcome after multivariable adjustment (OR, 1.001; 95% CI, 1.000–1.002; *P* = 0.161). Donor source was significantly associated with acute GVHD, with cord blood transplantation associated with a higher odds of acute GVHD compared with peripheral blood transplantation (OR, 5.663; 95% CI, 3.274–9.794; P < 0.001). HLA disparity was also significantly associated with acute GVHD (OR, 0.544; 95% CI, 0.296–0.998; *P* = 0.049).

**TABLE 4:** Logistic regression analysis of risk factors for death and acute GVHD in pediatric HSCT recipients.

Parameter	Death		Acute GVHD	
	OR (95% CI)	*P*	OR (95% CI)	*P*
Gender				
Female	Reference	​	Reference	​
Male	0.635 (0.221–1.828)	0.400	1.330 (0.710–2.491)	0.374
Age (years)	0.763 (0.452–1.288)	0.311	0.964 (0.804–1.155)	0.689
BW (kg)	1.021 (0.843–1.237)	0.831	0.983 (0.919–1.052)	0.619
Disease type				
Nonmalignant	Reference	​	Reference	​
Malignant	1.852 (0.192–17.867)	0.594	0.407 (0.105–1.582)	0.195
Donor source				
Peripheral blood	Reference	​	Reference	​
Cord blood	3.574 (1.181–10.817)	0.024	5.663 (3.274–9.794)	0.000
HLA disparity				
Mismatch	Reference	​	Reference	​
Match	0.473 (0.130–1.722)	0.256	0.544 (0.296–0.998)	0.049
AUC_0–6h_	1.002 (1.000–1.004)	0.069	1.001 (1.000–1.002)	0.161

### Dosing optimization

Monte Carlo simulations yielded an age- and body weight-stratified busulfan dosing regimen for administation every 6 h (Table 5). The probability of achieving the target AUC_0–6h_ of 900–1350 μM min varied substantially across age, body weight, and dose groups. Among infants aged 0–1 year, a dose of 1.0 mg/kg achieved the target attainment rates of 43.8% in those weighing 2.9–8.0 kg and 84.8% in those weighing 8.1–12.4 kg. Increasing the dose to 1.1 mg/kg increased target attainment to 87.5% and 100%, respectively, while a dose of 1.2 mg/kg achieved 100% target attainment in the 2.9–8.0 kg group. Among children aged 1.1–2.0 years, a dose of 1.1 mg/kg achieved the target attainment rates of 81.8% and 100% in patients weighing 5.9–9.0 kg and 9.1–13.2 kg, respectively. In the 9.1–13.2 kg group, target attainment was 50.0% with a dose of 1.0 mg/kg. For children aged 2.1–6.0 years, a dose of 1.1 mg/kg achieved target attainment in 89.3% of patients weighing 6.9–18.0 kg. Among those weighing 18.1–28.1 kg, target attainment increased from 46.7% with a dose of 0.95 mg/kg to 100% with a dose of 1.0 mg/kg. Among children aged 6.1–13.0 years, a dose of 1.0 mg/kg achieved target attainment in 78.3% of patients weighing 14.4–23.0 kg, whereas a dose of 0.95 mg/kg achieved target attainment in 95.1% of those weighing 23.1–34.0 kg. For patients weighing 34.1–55.3 kg, target attainment increased from 21.1% with a dose of 0.85 mg/kg to 100% with a dose of 0.9 mg/kg. Among adolescents aged 13.1–18.0 years, doses of 0.9 mg/kg and 0.95 mg/kg achieved target attainment in 57.1% and 100% of patients weighing 28.6–50.0 kg, respectively. In those weighing 50.1–70.0 kg, target attainment increased from 0% with a dose of 0.8 mg/kg to 100% with a dose of 0.85 mg/kg.

**TABLE 5:** Percentage of pediatric patients achieving target busulfan exposure with different dosing regimens.

Age (y)	BW (kg)	​	Target AUC_0–6h_ of 900–1350 μM⋅min					
		​	Dosage of busulfan (mg/kg)					
		0.8	0.85	0.9	0.95	1.0	1.1	1.2
0-1.0	2.9–8.0	-	-	-	-	43.8%	87.5%	100%
	8.1–12.4	-	-	-	-	84.8%	100%	-
1.1–2.0	5.9–9.0	-	-	-	-	-	81.8%	100%
	9.1–13.2	-	-	-	-	50.0%	100%	-
2.1–6.0	6.9–18.0	-	-	-	-	-	89.3%	100%
	18.1–28.1	-	-	-	46.7%	100%	-	-
6.1–13.0	14.4–23.0	-	-	-	-	78.3%	100%	-
	23.1–34.0	-	-	-	95.1%	100%	-	-
	34.1–55.3	-	21.1%	100%	-	-	-	-
13.1–18.0	28.6–50.0	-	-	57.1%	100%	-	-	-
​	50.1–70.0	0%	100%	-	-	-	-	-

The proposed closed-loop MIPD workflow is illustrated in Figure 5. Following an initial clinical assessment, the starting busulfan dose is determined according to the age- and body weight-stratified dosing regimen presented in Table 5. After drug administration, scheduled blood samples are collected and busulfan concentrations are quantified using HPLC-MS. AUC_0–6h_ is subsequently estimated. When the estimated AUC_0–6h_ falls within the target therapeutic window of 900–1350 μM min, the current dosing regimen is maintained. If exposure falls outside the target range, a pharmacist reviews the pharmacokinetic results and provides a dose-adjustment recommendation to the treating physician. The physician then modifies the subsequent busulfan dose accordingly. Because busulfan PK parameters may change during treatment, individual PK parameters estimated from the initial dose are not necessarily assumed to remain constant throughout the conditioning course. Therefore, intermediate PK reassessment may be incorporated when clinically indicated, particularly when observed exposure deviates substantially from the predicted target, when there are relevant changes in the patient’s clinical condition, or when there is concern about altered drug disposition. When additional PK data become available, the individual PK parameters can be re-estimated using the updated concentration data, and the subsequent busulfan dose can be adjusted accordingly, if necessary. This iterative process integrates therapeutic drug monitoring, longitudinal PK reassessment, re-estimation of individual PK parameters based on newly available concentration data, and model-guided dose adjustment, thereby allowing the initial first-dose PK assessment to guide early dose individualization while providing an opportunity to detect and respond to subsequent changes in busulfan disposition during treatment.

**FIGURE 5:**
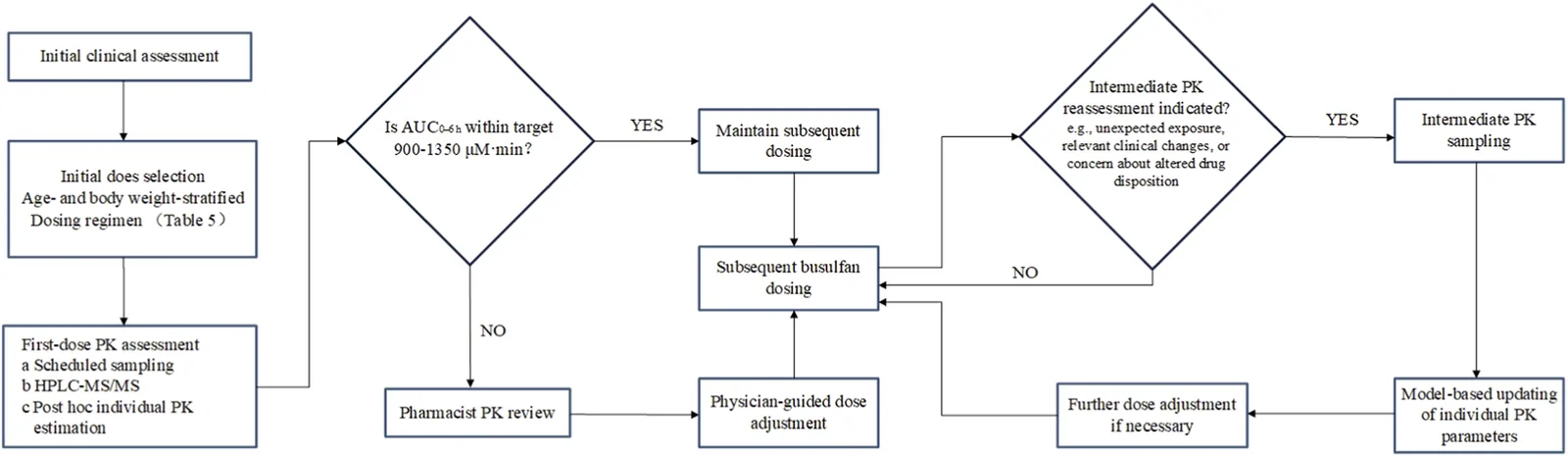
Model-informed precision dosing workflow for busulfan in pediatric allogeneic HSCT recipients.

## Discussion

In this prospective real-world study, busulfan pharmacokinetics in Chinese pediatric allogeneic HSCT recipients were adequately characterized by a two-compartment model with first-order elimination. Body weight and age were identified as important determinants of clearance, and the final model demonstrated good predictive performance in an internal evaluation cohort and robust individual-level predictive performance in LOOCV. Importantly, empirical dosing resulted in target exposure in only 47.6% of patients, highlighting the substantial variability in busulfan exposure under conventional dosing. In multivariable analysis, Busulfan AUC_0–6h_ was not significantly associated with mortalityor acute GVHD. Based on Monte Carlo simulations, we developed an age- and body weight-stratified initial dosing regimen and incorporated it into a closed-loop MIPD workflow integrating therapeutic drug monitoring and model-guided dose adjustment.

The estimated clearance for a 70-kg reference individual was 12.4 L/h, with a central volume of distribution of 40.8 L. The estimated clearance was broadly consistent with previously reported values in pediatric HSCT populations from Korea (10.7 L/h) (Rhee et al., 2017), the United Kingdom (12.2 L/h) (Solans et al., 2020), the United States (12.6 L/h) (Hoogd et al., 2020) and China (11.08 L/h) (Yuan et al., 2021). In contrast, the estimate was higher than that reported by Du et al. (7.71 L/h) (Du et al., 2022), despite broadly comparable patient characteristics. The estimated central volume of distribution was also comparable with values reported in two-compartment models developed in the United States (36.4 L) (Hoogd et al., 2020) and the United Kingdom (39.2 L) (Solans et al., 2020). The consistency of these estimates with several previously published models supports the structural plausibility of the present model. Although the two-compartment model provided a better fit to the observed data than the one-compartment model, as indicated by a lower OFV, model selection should also consider the practical requirements of clinical implementation. In pediatric HSCT, PK sampling may be sparse or occasionally incomplete because of clinical procedures, patient condition, or other practical constraints. Under such circumstances, a one-compartment model, with fewer structural parameters, may be more pragmatic and potentially more robust when only a limited number of informative samples are available. Importantly, a better population-level fit does not necessarily translate into more reliable individual parameter estimation under sparse or missing sampling conditions. In the present study, the two-compartment model was retained because it provided a better description of the observed concentration-time data and demonstrated satisfactory performance across multiple model evaluation procedures. Nevertheless, the two-compartment model should not be considered universally superior to a one-compartment model in all real-world sampling scenarios. Rather, its applicability within the proposed closed-loop MIPD workflow depends on the availability of sufficiently informative PK samples. A direct comparison of one- and two-compartment models under predefined sparse- and incomplete-sampling scenarios was beyond the scope of the present study. Future studies should therefore evaluate the relative robustness of these model structures for individual dose prediction and bedside MIPD implementation under different sampling conditions. Body size and maturation were significant determinants of busulfan disposition. Body weight has consistently been identified as an important covariate explaining interindividual variability in busulfan clearance in pediatric populations (Savic et al., 2013; Ishiwata et al., 2018; Long-Boyle et al., 2015; Solans et al., 2020; Hoogd et al., 2020; Veal et al., 2012; Paci et al., 2012). Allometric scaling provides a physiologically plausible approach for accounting for body-size-related differences in drug disposition and has been widely incorporated into pediatric busulfan models (Hoogd et al., 2020; Veal et al., 2012; Neroutsos et al., 2022; Anderson and Holford, 2009). Accordingly, we incorporated body weight into the clearance model using a fixed allometric exponent of 0.75. Age was independently associated with clearance after accounting for body weight. Although postmenstrual age (PMA) may theoretically provide a more physiologically appropriate measure of maturation than postnatal age (PNA), gestational age data were unavailable in our dataset. Therefore, PNA was used as the maturation descriptor. In the present model, busulfan clearance reached approximately 50% of the mature value at a PNA of 0.217 years, corresponding to approximately 11.3 weeks of PNA. Assuming a gestational age of 37 weeks, this corresponds to approximately 48.3 weeks of PMA. This estimate was broadly comparable to previously reported TM_50_ values based on PMA (45.7 weeks (McCune et al., 2014) and 31 weeks (Du et al., 2022)). Differences in the maturation descriptor and patient populations may partly explain these discrepancies. After incorporation of body weight and age, no additional covariates significantly improved the model. Although hepatic and renal function may theoretically affect busulfan disposition, ALT, AST, serum creatinine, and creatinine clearance were not significant predictors of clearance in our cohort. Similarly, although previous studies have suggested that concomitant fludarabine administration may reduce busulfan clearance (Ishiwata et al., 2018; Castro et al., 2013), we did not identify a significant effect of fludarabine coadministration. These findings suggest that the major sources of between-subject variability in our population were adequately captured by body size and developmental maturation.

The relationship between busulfan exposure and clinical outcomes remains incompletely defined. Previous studies have yielded inconsistent findings regarding the association between busulfan exposure and mortality. Bremer et al. reported that higher first-dose busulfan exposure was associated with increased 3-month mortality in Caucasian adolescents and adults (Bremer et al., 2015), while Bartelink et al. reported higher treatment-related mortality among patients with high busulfan exposure (Bartelink et al., 2016). In contrast, Davis et al. did not identify a significant association between first-dose exposure and mortality (Davis et al., 2019), and Takachi et al. found no significant relationship between busulfan pharmacokinetics and survival (Takachi et al., 2019). In the present study, higher busulfan AUC_0–6h_ was not independently associated with mortality after adjustment for relevant clinical covariates, although the point estimate suggested a trend toward higher mortality risk (OR, 1.002; 95% CI, 1.000–1.004; P = 0.069). This finding should therefore be interpreted cautiously. The relationship between busulfan exposure and acute GVHD is similarly controversial. Some studies have reported increased rates of GVHD at higher busulfan exposure (Bartelink et al., 2016; Ansari et al., 2017), whereas others have suggested an association between lower exposure and increased acute GVHD risk (Russell et al., 2013). Conversely, several pediatric studies have not identified a significant association between busulfan exposure and grade II-IV acute GVHD (Cao et al., 2025; Baker et al., 2000; Shao et al., 2022). Consistent with these latter findings, our multivariable analysis did not demonstrate an independent association between AUC_0–6h_ and acute GVHD. Although AUC_0–6h_ was higher among patients who developed acute GVHD in the unadjusted analysis, this association did not remain statistically significant after adjustment for donor source and HLA disparity. These findings emphasize the multifactorial nature of GVHD and suggest that busulfan exposure alone may not be a major determinant of acute GVHD in this population. Taken together, these findings do not provide sufficient evidence to establish or redefine the optimal therapeutic exposure range for busulfan in Chinese pediatric HSCT recipients. The conventional target range of 900–1350 μM min used in this study was adopted as a reference target for Monte Carlo simulation and initial dose optimization rather than as a prospectively validated therapeutic window in this population. The lack of a statistically significant association between first-dose AUC_0–6h_ and mortality or acute GVHD should therefore not be interpreted as evidence against this target range, but rather highlights the need for prospective studies specifically designed to define exposure-efficacy and exposure-toxicity relationships and to establish population-specific therapeutic targets.

The inability of weight-based dosing to consistently achieve target exposure provides a strong rationale for model-based initial dosing. Weight-based dosing has been associated with higher incidence of sinusoidal obstruction syndrome (41.7% vs. 5.4% with age-based dosing; HR: 9.46, P = 0.009) (Gökçebay et al., 2015). In our cohort, only 47.6% of patients achieved the target AUC_0–6h_ following empirical weight-based dosing, while nearly half had subtherapeutic exposure. Our simulation-based dosing strategy integrates age and body weight into a clinically simple dosing table. The FDA and EMA currently recommend weight-based dosing strategies, although the dose categories differ between regulatory frameworks. The FDA recommends 1.1 mg/kg for patients weighing ≤12 kg and 0.8 mg/kg for those weighing >12 kg, whereas the EMA recommends doses ranging from 0.8 to 1.2 mg/kg across five weight categories (<9 kg: 1.0 mg/kg; 9–16 kg: 1.2 mg/kg; 16–23 kg: 1.1 mg/kg; 23–34 kg: 0.95 mg/kg; >34 kg: 0.8 mg/kg). Our simulations suggested that additional age stratification within weight categories may improve target attainment, particularly in younger children. For infants weighing 2.9–8.0 kg, target attainment increased from 43.8% with a dose of 1.0 mg/kg to 87.5% with 1.1 mg/kg and reached 100% with 1.2 mg/kg. In infants weighing 8.1–12.4 kg, target attainment increased from 84.8% with 1.0 mg/kg to 100% with 1.1 mg/kg. Among children aged 1–13 years, the simulated optimal dose decreased progressively with increasing body weight. Adolescents similarly demonstrated a lower dose requirement at higher body weights. These findings support the integration of maturation with body-size scaling when selecting an initial busulfan dose.

The proposed workflow may also facilitate multidisciplinary implementation. In our framework, physicians select the initial dose using the age- and body weight-stratified regimen, while pharmacists interpret busulfan concentration data and model-derived AUC_0–6h_ estimates and provide dose-adjustment recommendations. Such pharmacist-physician collaboration creates an iterative feedback loop between drug administration, exposure assessment, and dose adjustment. Importantly, subsequent PK reassessment can be undertaken when clinically indicated, with newly obtained concentration data used to re-estimate individual PK parameters and inform subsequent dose adjustments. This approach may be particularly valuable in pediatric HSCT, where rapid developmental changes and substantial pharmacokinetic variability make fixed dosing strategies inherently limited.

Several limitations should be acknowledged. First, this was a single-center study, which may limit the generalizability of the model to other institutions and pediatric populations. Nevertheless, the relatively large cohort and broad range of ages and body weights provided substantial information for model development. Although a truly independent external dataset was unavailable, we performed internal evaluation using a held-out cohort and further assessed model robustness using LOOCV. These analyses provided supportive evidence of the model’s predictive performance, but external validation in an independent population remains necessary. Multicenter external validation will be necessary to determine whether the model and dosing regimen can be generalized to other Chinese and international pediatric HSCT populations. An additional limitation concerns the interpretation of the peripheral volume of distribution (V_2_). In the final model, V_1_ was described using a linear body-weight relationship and was estimated as 40.8 L for a 70-kg reference individual, whereas V_2_ was estimated as 1.27 L without a body-weight covariate. Although the two-compartment structure provided a substantially better statistical description of the observed concentration-time data than the one-compartment model, the relatively small V_2_ should be interpreted cautiously and should not be considered a directly measurable physiological peripheral volume. The available sampling design, particularly the limited sampling during the early distribution phase, may also have constrained the precision with which the distributional component could be characterized. Therefore, the physiological interpretation of V_2_ remains uncertain, and further studies with richer early distribution-phase sampling are warranted to confirm the robustness of the two-compartment structure. Second, the TM50 parameter, which characterizes the midpoint of age-related maturation in busulfan clearance, was estimated with relatively limited precision. The RSE of TM50 was 36.6%, and the bootstrap 95% CI was 0.107–2.18 years. This uncertainty may introduce some imprecision into the characterization of maturation and, consequently, into age-dependent dose predictions, particularly in younger children. However, TM50 represents the midpoint of a continuous maturation function rather than a discrete clinical age threshold. Therefore, the proposed age-stratified dosing regimen should be interpreted as model-informed dosing guidance rather than as rigid age-based dosing rules. Further evaluation in larger and independent pediatric cohorts, particularly among younger children, is warranted to confirm the robustness of the maturation component and its implications for dose optimization. Third, GST genetic polymorphisms were not evaluated in the present analysis. Genetic variation in GSTA1 and GSTP1 has been investigated as a potential determinant of busulfan clearance, although its clinical contribution has not been consistently demonstrated across studies (Zwaveling et al., 2008; Sun et al., 2020). Our previous work suggested that GST activity may contribute to busulfan pharmacokinetic variability (Cao et al., 2025). Therefore, incorporating pharmacogenetic information into future model development may further improve individualized dosing, particularly in patients whose exposure remains poorly predicted by demographic covariates alone. Finally, the exposure-outcome analysis was observational and based primarily on mortality and acute GVHD. Although higher AUC_0–6h_ showed a trend toward an association with mortality, the association did not reach conventional statistical significance, and residual confounding cannot be excluded. The findings should not be interpreted as establishing a causal exposure threshold for mortality. Prospective multicenter studies are warranted to determine whether exposure-guided MIPD improves clinically relevant outcomes while reducing busulfan-related toxicity.

## Conclusion

In this prospective real-world cohort of Chinese pediatric allogeneic HSCT recipients, busulfan pharmacokinetics were adequately described by a two-compartment model incorporating body weight and age as key determinants of clearance. This framework provides a practical approach to individualized busulfan dosing and may facilitate the implementation of precision dosing in pediatric allogeneic HSCT. Prospective multicenter validation is warranted to further evaluate its generalizability and clinical utility.

## Data Availability

The original contributions presented in the study are included in the article/supplementary material, further inquiries can be directed to the corresponding authors.
